# Perceiving Control Over Aversive and Fearful Events Can Alter How We Experience Those Events: An Investigation of Time Perception in Spider-Fearful Individuals

**DOI:** 10.3389/fpsyg.2012.00337

**Published:** 2012-09-17

**Authors:** Simona Buetti, Alejandro Lleras

**Affiliations:** ^1^Department of Psychology, University of Illinois at Urbana–ChampaignChampaign, IL, USA

**Keywords:** feeling of control, time perception, spider-fearful, emotion and cognition, emotion regulation

## Abstract

We used a time perception task to study the effects of the subjective experience of control on emotion and cognitive processing. This task is uniquely sensitive to the emotionality of the stimuli: high-arousing negative stimuli are perceived as lasting longer than high-arousing positive events, while the opposite pattern is observed for low-arousing stimuli. We evaluated the temporal distortions of emotionally charged events in non-anxious (Experiments 1 and 5) and spider-fearful individuals (Experiments 2–4). Participants were shown images of varying durations between 400 and 1600 ms and were asked to report if the perceived duration of the image seemed closer to a short (400 ms) or to a long (1600 ms) standard duration. Our results replicate previous findings showing that the emotional content of the image modulated the perceived duration of that image. More importantly, we studied whether giving participants the illusion that they have some control over the emotional content of the images could eliminate this temporal distortion. Results confirmed this hypothesis, even though our participant population was composed of highly reactive emotional individuals (spider-fearful) facing fear-related images (spiders). Further, we also showed that under conditions of little-to-no control, spider-fearful individuals perceive temporal distortions in a distinct manner from non-anxious participants: the duration of events was entirely determined by the valence of the events, rather than by the typical valence × arousal interaction. That is, spider-fearful participants perceived negative events as lasting longer than positive events, regardless of their level of arousal. Finally, we also showed that under conditions of cognitive dissonance, control can eliminate temporal distortions of low arousal events, but not of high-arousing events, providing an important boundary condition to the otherwise positive effects of control on time estimation.

## Introduction

It is agreed that the feeling of control that we experience over external events in the world and over our behaviors, thoughts, emotions, cognitions and beliefs, fosters mental health, physical health, and is hence crucial for evolutionary survival (e.g., Shapiro et al., 1996). This feeling of wellbeing arises from a complex interaction involving psychological, psycho-sociological, and biological factors, that altogether foster mental health, physical health, and psychosocial functioning (Lefcourt, 1973; Langer and Rodin, 1976; Bandura, 1977; Thompson, 1981; Burger, 1985; Wiedenfeld et al., 1990; Armfield and Mattiske, 1996; Shapiro et al., 1996; Bandura et al., 2003; Leotti et al., 2010; Allman and Meck, 2012). Importantly, this subjective experience of control is not necessarily related to an actual causal control over the world (Langer, 1975). A consistent finding across different species (e.g., humans, non-human primates, rats, dogs, for a review, see Lefcourt, 1973) is that the perception of control can alter the aversive quality of an external stressor (e.g., Glass et al., 1969). Recently, it was suggested that the perception of control plays a critical role in regulating the emotional response triggered by environmental stressors, in such a way that it buffers our emotional reactions to aversive stimuli (Leotti et al., 2010; Mereu and Lleras, in press). In the present report, we tested this hypothesis in a highly reactive emotional group of participants (sub-clinical spider-fearful individuals) within the context of a time perception task. Because it is well known that the emotional content of an event influences how long we experience that event (e.g., Angrilli et al., 1997; Droit-Volet and Meck, 2007; Smith et al., 2011), we evaluated whether having a feeling of control over emotional and fear-related events altered the manner in which those events were experienced.

### Experiencing illusory control over external events

People like to believe that their choices or actions affect the events around them, even in uncontrollable situations (a phenomenon referred to as the illusion of control by Langer, 1975; see also Presson and Benassi, 1996 for a meta-analytical review). For example, one may push several times the elevator “call” button and as a result may feel that the elevator arrived sooner because of one’s actions. This feeling is also evidenced in superstitious behavior (e.g., Skinner, 1948). A feeling of control can be experienced when outcomes (i.e., events in the world) are entirely independent of one’s choices, provided that the person concerned is sufficiently motivated to achieve the outcome and that the desired outcome occurs often (Jenkins and Ward, 1965; Alloy and Abramson, 1979; Thompson, 1999). For instance, in an experiment a person may be given a choice between two keys (key 1 or 2) and one of two outcomes (happy or sad face) may occur after their keypress. Imagine a situation in which participants are asked to press buttons 1 and 2 in such a way as to maximize the occurrence of happy faces. If a happy face appears 75% of the time, *irrespective of the button chosen*, participants will report having had control (though not perfect) over the outcome. If on the other hand, a happy face occurs rarely (e.g., 25% of the time), they will report having little-to-no control over the events. This belief that one’s actions impact the world in probabilistic or deterministic fashion is very common (Taylor and Brown, 1994; Presson and Benassi, 1996; Thompson, 1999), and occurs as soon as one is given the opportunity to exert a choice and is put in a position of being an active agent in a situation (Langer, 1975; Langer and Rodin, 1976; Thompson, 1999; Leotti et al., 2010). Taylor and Brown (1988, 1994) considered the illusion of control as being a “positive” illusion (or cognitive bias) that promotes individual’s wellbeing by fostering, for instance, good copying strategies, high motivation in various aspect of the life, high productivity, and positive social exchanges.

### Benefits of perceived control over external stressors

A feeling of control over an external stressor has substantial beneficial effects on the period preceding the occurrence of the external stressor (Stotland and Blumenthal, 1964; Geer et al., 1970; Geer and Maisel, 1972; Gatchel and Proctor, 1976). For instance, participants that were provided with a feeling of control showed decreased physiological arousal and reported less discomfort and less anxiety compared to participants that were not given a feeling of control in the period preceding the administration of a painful stimulation (e.g., electric shocks, loud tones, Geer et al., 1970; Gatchel and Proctor, 1976), the presentation of high-arousing negative images (Geer and Maisel, 1972), and the administration of a series of tests (Stotland and Blumenthal, 1964). See Thompson (1981) for a review of different methods used to study the effects of perceived control on aversive stimulation.

Most remarkable, compared to participants that were not given any feeling of control over stressful situations, participants provided with a feeling of control over the stressors showed better performance on later cognitive tasks (puzzle solving and proofreading tasks) that did *not* involve any manipulations of control nor any stressors (Glass et al., 1969). This type of long-lasting benefits of perceived control over stressful events has also been shown in animals. In rats, experiencing heightened levels of control during aversive events can increase emotional resilience to future social-stressors that are themselves uncontrollable. For example, rats that learned to turn the wheel for a specific amount of turns to escape a series of electric shocks and that 7 days later were faced with social-stress (i.e., confronted with an alpha male rat) handled the stress much like rats that were never electrocuted: they showed a normal rate of defeat/submission behaviors. In comparison, rats that were not given the possibility to escape the electric shocks showed an abnormal increase of defeat/submission behaviors when facing the alpha male rat (Amat et al., 2010). Finally, studies on monkeys showed that control helps to reduce the negative lasting impact of previous stressors on physical health (Weiss, 1971). Thus, experiencing a sense of control over a potentially traumatic event (electric shock) can provide a measure of psychological resilience to future stressful events and diminish the sequelae of those events.

It is important to note that while the feeling of control seems to have a strong influence on the period preceding the aversive event, there are conflicting results as to whether it can actually alter the perception of the aversive event itself: both increases and decreases in physiological arousal in response to the aversive event have been found when control was increased (e.g., Corah and Boffa, 1970; Geer et al., 1970; Geer and Maisel, 1972; Gatchel and Proctor, 1976) and no benefit has been observed in the overall estimation of pain and stress (e.g., Pervin, 1963; Stotland and Blumenthal, 1964; Glass et al., 1969). That said, participants that are given a feeling of control over a painful stimulus are willing to endure more painful stimulation and show higher pain tolerance than those deprived of a sense of control (e.g., Glass et al., 1969).

In sum, research that evaluated the impact of perceived control on external stressors indicates that some aspects of the aversive quality of the stimulus can be decreased by providing participants with a feeling of control and the possibility to exert choice. This indicates that the feeling of control may play an important role in the cognitive reappraisal of aversive events. As proposed by Leotti et al. (2010), we believe that having a sense of control over the aversive events can provide a buffering effect on the otherwise strong impact of emotion on our cognitive processes (see also Mereu and Lleras, in press).

### Goal of the present study

Here, we studied the impact of perceived control on one perceptual aspect of the aversive stimulation: its duration. We chose to study time perception because it provides us with an experimental platform to test the effects of the feeling of control on a cognitive process that is uniquely sensitive to the emotionality of the event being judged (e.g., Angrilli et al., 1997; Smith et al., 2011). Specifically, when judging the duration of high-arousing events, people perceive negative events (e.g., images of dismembered bodies) as lasting longer than positive events (e.g., erotic images). When judging the duration of low-arousing events, people perceive negative events (e.g., a dirty mop) as lasting shorter than positive events (e.g., a pretty flower). In sum, time perception shows sensitivity to both arousal and valence in a very distinct pattern, which we can now leverage to study the impact of perceived control on emotional processing. Moreover, the study of time perception is interesting on its own right because these emotion-induced time distortions can be frequently experienced in everyday life. For example, the time spent waiting for a loved one seems longer than the one spent with them. We also frequently experience daily events as lasting overly long, when we are in a hurry and there are obstacles in our way (e.g., traffic lights seem to take overly long to turn to green). The goal of our research is to study whether the subjective feeling of control can alter these temporal distortion effects. The rationale is that, if a feeling of control can buffer our cognition from our emotional reactions to emotional events (see Leotti et al., 2010; Mereu and Lleras, in press), then performance on the time estimation task (a cognitive task) will no longer be influenced by the emotionality of the images themselves.

Here we use a time bisection task (Penney et al., 2008) to evaluate temporal distortions elicited by emotional stimuli. In a time bisection task, participants are initially taught to discriminate between two standard durations (one short and one long). Then they are shown events of varying duration and asked to judge whether the duration of the event is closer to the short or to the long standard. This procedure allows one to estimate a psychophysical curve relating the real duration of events to participants’ perceived duration of that event (by plotting the likelihood that a given event is perceived as more similar to the long standard). One can compute separate psychophysical curves for different types of events and thus estimate whether time is perceived differently across those different types of events. The key measure to compare these curves is the bisection point (BP): the point at which participants are equally likely to report an event as being more similar to the short or long standards. Let’s take as examples high-arousing positive images and high-arousing negative images. Typically, high-arousing negative images are perceived as lasting longer than high-arousing positive images. In the psychophysical curve, this means that for any given event duration, participants are more likely to respond long for negative compared to positive high-arousing images. Therefore, the psychophysical curve for the negative images is shifted to the left compared to that for the positive images. Consequently, the BP for negative images is smaller than the one for positive images. In other words, a smaller BP indicates an earlier transition from events being perceived as short to events being perceived as long, and therefore indicates an overestimation of time in that condition.

Mereu and Lleras (in press) first tested the impact of perceived control on the duration of high-arousing emotional events by using images from the International Affective Picture System (IAPS images, Lang et al., 2008). Their results showed that under a condition of low perceived control, participants evaluated negative events as lasting longer than positive events (replicating Angrilli et al.’s, 1997 results), whereas participants under conditions of high perceived (illusory) control experienced no temporal distortions. Here, we aim to extend those initial findings in two important directions: (1) we wanted to perform a stronger test of our hypothesis by investigating whether this buffering effect of control on time perception would also be observed in a highly emotionally reactive population: spider-fearful participants confronted with spider images; (2) we wanted to determine some of the boundaries of this effect.

The overall structure of the paper is as follows. Experiment 1 replicates the temporal distortion effects typically observed in non-anxious individuals, deprived of control, when faced with emotional images, including a subset of spider images. We used these results as a benchmark. Experiment 2 tested sub-clinical spider-fearful individuals, also deprived of control, on the same set of images. We observed large temporal distortions driven by emotional content, though the pattern of temporal distortions was different from that of non-anxious individuals. Experiment 3 was our critical experiment in which we tested the influence of high levels of control on time perception in this highly reactive population. No time distortion effects were observed. Experiment 4 provided an experimental control condition in which spider-fearful individuals experience the same images as those in Experiment 3, but were once again, deprived of any feeling of control. Time distortions were again evident, replicating Experiment 2. Finally, Experiment 5 tested whether high levels of perceived control, alone, are sufficient to eliminate time distortion effects. They are not: in a condition of high cognitive dissonance, non-anxious participants showed temporal distortions of high-arousing events.

## Experiment 1: 25% of Positive Images – Non-Anxious Individuals

We used a time bisection task to evaluate time distortion effects in non-anxious individuals *under conditions of low experienced control over the events*. Participants were shown images that varied along their arousal and valence dimensions. According to previous studies (Jenkins and Ward, 1965; Alloy and Abramson, 1979), the subjective experience of control can be diminished by reducing the occurrence of a desired outcome. Thus, in Experiment 1, we presented a high percentage of negative pictures (75%) and instructed participants to try to *minimize* the occurrence of negative images by selecting one of two buttons at the beginning of each trial. Importantly, the occurrence of pleasant and unpleasant images was entirely independent of participants’ choices. Thus, the sense of control over the events was not real, just illusory, and given the low percentage of positive images, we anticipated participants experiencing low levels of control over the emotionality of the pictures.

The aim of this first experiment was to provide a replication of previous findings in the time perception literature (Angrilli et al., 1997; Smith et al., 2011) by using a new subset of pictures (spiders) in addition to images taken from the IAPS (Lang et al., 2008) in a group of normal, non-anxious, and non-spider-fearful individuals. The IAPS pictures were categorized into three sets: high-arousing positive, high-arousing negative, and low-arousing positive. The spider images were expected to be judged as being low-arousing and slightly negative or neutral by non-anxious individuals (see Buetti et al., 2012). Thus, we expected that non-anxious participants would overestimate the duration of high-arousing negative images compared to arousal-matched positive images (i.e., BP for negative images will be smaller than for positive images). Conversely, for low-arousing images, positive pictures should be perceived as lasting longer than spider images (i.e., BPs for positive images will be smaller than for negative images).

### Methods

#### Participants

Participants were students at the University of Illinois. Sixteen non-anxious individuals (14 females, 2 males, mean age of 20.8 years, 1.9 of mean at the Fear of Spiders Questionnaire, FSQ) participated in the study in exchange for one psychology course credit. They were selected on the basis of their scores at the FSQ (Szymanski and O’Donohue, 1995). All participants were naïve regarding the selection criteria and questionnaires scores, and had normal or corrected-to-normal vision. The study was approved by the university IRB board.

#### Stimuli and design

We used two types of stimuli to train participants to discriminate between two standard durations, that is, a short (400 ms) and a long (1600 ms) duration. First, short and long durations alternated across trials (*N* = 8) and participants reported the perceived duration of a pink oval displayed on the center of the screen. Second, short and long durations were randomized and participants reported the perceived duration of eight IAPS neutral pictures (on both arousal and valence dimensions). In this second training task, participants were given feedback about their performance and they were required to reach 100% of correct answers before continuing to the next task.

In the *time bisection task*, we used seven stimulus durations (400, 600, 800, 1000, 1200, 1400, and 1600 ms) and participants were asked to report if the perceived duration was more similar to the short or to the long standard duration. The stimuli consisted in four sets of pictures (25.5° × 21°). Three sets were chosen from the IAPS: low-arousing positive pictures (arousal score: 3.8, valence score: 7.1), high-arousing positive pictures (6.5, 6.5) and high-arousing negative pictures (6.2, 2.0). The fourth set consisted of spider pictures^1^ that were taken from the internet and that were evaluated by the participants at the end of the experiment using the same method used to rate the IAPS pictures (Self-Assessment Manikin, Lang, 1980). This method allowed us to obtain three mean scores describing the level of arousal, valence, and domination experienced by participants when confronted with our set of spider pictures. Responses were given on a 1–9 scale and scores were recoded so that low and high scores on the *arousal dimension* indicate low and high levels of arousal, respectively; on the *valence dimension*, low and high scores refer to negative and positive valence, respectively; and on the *dominance dimension*, low and high scores indicate that participants felt controlled or in control when confronted to the spider pictures.

For the time bisection task, we chose 8 IAPS low-arousing positive pictures, 8 IAPS high-arousing positive pictures, 24 IAPS high-arousing negative pictures, and 24 spider pictures. See Appendix for a list of the IAPS images used. In order to better estimate the BP in the psychophysical curve, all pictures were shown for each of the three central time durations (800, 1000, and 1200 ms). To diminish the number of trials in the session, we reduced the number of pictures for the two shortest (400 and 600 ms) and the two longest (1400 and 1600 ms) time durations. Indeed, long and short categorization was much easier for those time durations compared to the three central time durations. Table 3 provides the actual number of trials per condition, for Experiments 1–5.

Overall, across the whole experiment, participants completed a total of 352 trials, presented in a random order. All stimuli were displayed on a white background at the center of the screen.

#### Procedure

The experimental session contained the following tasks. First, participants completed three questionnaires in the following order: Short Depression-Happiness Scale (SDHS, Joseph et al., 2004), short form of the State Anxiety Inventory (s-STAI, Marteau and Bekker, 1992), FSQ (Szymanski and O’Donohue, 1995). Second, they were trained to discriminate between short and long standard durations, first with the pink oval stimulus and then with IAPS neutral pictures. The training trials started with a black fixation cross displayed on the centre of the screen for a random duration, varying between 400 and 900 ms. Then, the training-stimulus was presented for 400 or 1600 ms. Three-hundred milliseconds later, the message “Was the duration of the oval/image short or long?” was shown and the participants responded “short” and “long” by pressing one of two buttons (key 4 or 6), that were counterbalanced across participants. Visual feedback (“Correct” or “Wrong answer”) was provided in the training trials with the neutral IAPS images.

**Figure 1:**
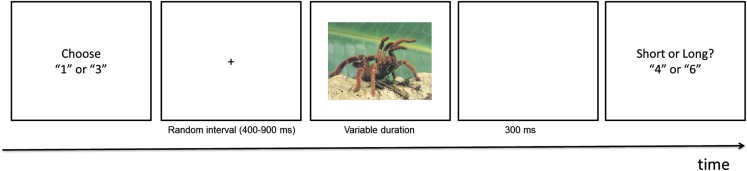
**Schematic of the trial events in the experiments**. Trials started with the choice display where participants had to choose either the “1” or “3” key in order to try to influence the valence of the subsequent image. The display remained on the screen until a choice was recorded. A fixation display was then presented for a random interval anywhere between 400 and 900 ms. This variable interval was followed by the presentation of the emotional picture which was presented for one of seven possible durations (400, 600, 800, 1000, 1200, 1400, and 1600 ms). The image then was replaced by a blank screen for 300 ms. Then participants were prompted to categorize the image duration as short of long on the final display.

Third, participants performed the *time bisection task* that included a manipulation aimed to induce a sense of control at the beginning of each trial (see Figure 1). Participants were asked to choose between one of two keys (1 and 3) to try to *decrease the occurrence of negative images*: they were instructed to try to find out different combinations of keypresses that would yield a high rate of positive images. Across all experiments, some participants failed to comply with these instructions (e.g., they pushed just one key along the whole experiment), and those were not considered for further analysis. Importantly, there was no contingency between the selection of one of the two keys and the outcome. Because in this experiment negative pictures occurred on 75% of the trials, we expected participants to experience a low level of control over the image content (i.e., the valence). Each trial started with the message “Make your choice: 1 or 3” and once one of the two keys was pressed, the fixation cross was presented for a random interval between 400 and 900 ms. Afterward, an image was displayed for one of the seven time durations (400, 600, 800, 1000, 1200, 1400, and 1600 ms) and 300 ms after the image disappeared, the message “Was the duration of the image short or long?” was presented. Participants were asked to judge whether the duration of the presented image was more similar to the duration of the short or long standard interval by pressing one of two keys (4 or 6). A 500 ms blank screen preceded the next trial. Participants completed 8 blocks of 44 trials, for a total of 352 trials, with a pause after each block.

At the end of the time bisection task, participants were asked to respond to the following questions (in order) by means of a continuous scale going from 0 to 100%: (1) how often did positive images appear?; (2) Did you feel at any point of the experiment that you had control over the images?; and (3) How liberal are you? Then, they completed the s-STAI a second time. Afterward, they completed the Desirability of Control DC Scale (Burger and Cooper, 1979) and finally, they rated the valence, arousal, and domination for each of the spider pictures by using the nine-point scale of the Self-Assessment Manikin (Lang, 1980). The entire session lasted about 50 min.

### Results

#### Questionnaires and evaluation of the spider pictures

Scores from the different questionnaires are reported in Table 1. Overall, non-anxious participants were not afraid of spiders (FSQ = 1.9), had relatively low state anxiety levels that did not significantly vary between the first and second assessment [STAIs mean before and after the bisection task: 11.5 and 11.9; *t*(15) = −0.32, *p* = 0.75], and were not at risk for depression (SDQ = 13). At the end of the time bisection task, non-anxious participants reported that positive images occurred in 29% of the trials. They considered themselves as being relatively liberal (67%). More importantly, they reported experiencing very low levels of control over the image content (13%).

**Table 1:** **Mean scores (standard errors in parentheses) from the experimental questions and questionnaires completed in Experiments 1–5**.

	Experiment 1 (*N* = 16)	Experiment 2 (*N* = 16)	Experiment 3 (*N* = 16)	Experiment 4 (*N* = 16)	Experiment 5 (*N* = 16)
FSQ	1.9 (0.2)	5.6 (0.2)	5.6 (0.2)	5.1 (0.2)	2.1 (0.2)
SDQ	12.8 (0.7)	13.3 (0.7)	14.4 (0.7)	13.4 (0.9)	13.6 (0.8)
STAIs-pre	11.5 (0.9)	12.7 (0.9)	10.4 (0.9)	11.8 (1.2)	11.3 (1.1)
STAIs-post	11.9 (0.7)	15.8 (1.1)	13.3 (0.9)	16.5 (1.1)	12.9 (1.0)
DC	93.1 (2.9)	94.9 (3.5)	98.2 (3.5)	99.8 (3.4)	96.1 (0.4)
% Positive images	28.9 (2.2)	28.2 (2.7)	62.8 (4.4)	63.4 (2.8)	26.1 (2.9)
% Participant in control	12.6 (3.7)	16.1 (4.1)	53.9 (4.4)	11.2 (2.8)	54.6 (5.6)
% Computer in control	–	–	–	85.5 (3.5)	–
% Liberal	67.2 (6.2)	67.2 (6.7)	66.2 (5.0)	63.5 (5.2)	65.9 (5.1)

*FSQ, Fear of Spider Questionnaire; SDQ, Short Depression-Happiness Scale; STAIs-pre: short form of the State Anxiety Inventory filled out before the Time Bisection Task; STAIs-post: short form of the State Anxiety Inventory filled out after the Time Bisection Task; DC, Desirability of Control Scale; % Positive images: participants were asked to report how often did positive images appear in the experiment?; % Participant in control: participants were asked to report if they felt that at any point in the experiment they felt that they had control over the emotional content of the images; % Computer in Control: participants were asked to report how much the computer choice influenced the image content (only assessed in Experiment 4); % Liberal: participants were asked to report the extent to which they estimated themselves as being liberal*.

In the *evaluation of the spider pictures*, the means observed for the three dimensions of the Self-Assessment Manikin scale, arousal, valence, and dominance, were 3.8, 5.2, and 5.4, respectively (see Table 2). Thus, non-anxious participants considered spider pictures as being low-arousing and neutral in valence. Because spider pictures were not considered as being positive in valence by non-anxious participants, for these participants, 25% of images were highly positive. Notice that participants were quite accurate at reporting this frequency when they estimated the percentage of positive images at the end of the experiment (29%)^2^.

**Table 2:** **Mean arousal and valence (standard deviation shown in parenthesis) of the four image sets used in Experiments 1–5**.

Image set	Dimension	Experiment 1	Experiment 2	Experiment 3	Experiment 4	Experiment 5
IAPS high-arousing positive	Arousal	6.5 (2.1)	6.5 (2.1)	6.5 (2.1)	6.5 (2.1)	6.5 (2.1)
	Valence	6.5 (1.9)	6.5 (1.9)	6.8 (1.8)	6.8 (1.8)	6.5 (1.9)
IAPS high-arousing negative	Arousal	6.2 (2.3)	6.2 (2.3)	6.3 (2.4)	6.3 (2.4)	6.2 (2.3)
	Valence	2.0 (1.3)	2.0 (1.3)	1.9 (1.3)	1.9 (1.3)	2.0 (1.3)
IAPS low-arousing positive	Arousal	3.8 (2.2)	3.8 (2.2)	3.8 (2.2)	3.8 (2.2)	3.8 (2.2)
	Valence	7.1 (1.4)	7.1 (1.4)	7.0 (1.5)	7.0 (1.5)	7.1 (1.4)
Spider pictures	Arousal	3.8 (1.2)	6.5 (2.1)	6.7 (0.4)	7.4 (0.3)	3.9 (0.3)
	Valence	5.2 (1.4)	2.7 (1.4)	1.8 (0.2)	2.2 (0.4)	4.8 (0.3)

#### Time bisection task

Temporal judgments for each set of images were examined by calculating the BPs from the individual psychometric functions obtained after running a logistic regression. The BP represents the duration at which the psychophysical curve crosses the “50% Long” likelihood. To test for temporal distortion effects we ran two separate ANOVAs on BPs with Valence as within-subjects factors and compared arousal-matched conditions (IAPS high-arousing positive images vs. IAPS high-arousing negative images; and IAPS low-arousing positive images vs. low-arousing neutral spider images).

**Figure 2:**
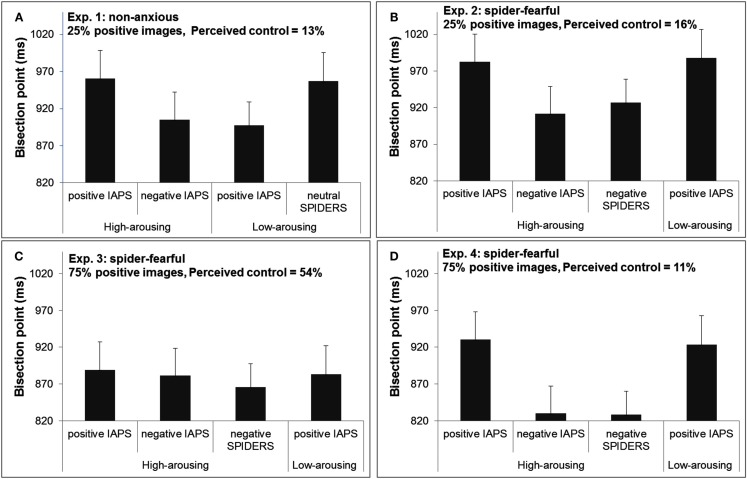
**Bisection points in Experiments 1–4 [(A–D), respectively], as a function of emotional content of image events**. Note that when comparing two bisection points, the smaller bisection point corresponds to images in that category having been perceived as lasting longer than the images with a larger bisection point. Error bar indicates the standard error of the means.

The results indicated that for high-arousing stimuli, negative pictures (BP = 905 ms) were perceived as lasting longer than positive pictures (BP = 961 ms), *F*(1,15) = 9.47, *p* < 0.01, while for low-arousing stimuli, positive stimuli (BP = 897 ms) were perceived as lasting longer than neutral stimuli (BP = 957 ms), *F*(1,15) = 5.46, *p* < 0.05 (see Figure 2A).

### Discussion

In Experiment 1 we tested non-anxious, non-spider-fearful participants to measure time distortion effects for different sets of emotional pictures under conditions of low level of experienced control, including a set of spider images. Including a set of spider images was important to establish a baseline to compare their performance to that of spider-fearful individuals (Experiment 2). As expected, with a high proportion of negative pictures (75%) and the instruction of reducing the proportion of negative images, participants experienced very low feelings of control over the events in the experiment (13%). Importantly, under this condition of low level control, we successfully replicated previous findings from the literature with non-anxious individuals for whom the perceived duration of emotional events is determined by both arousal and valence (Angrilli et al., 1997; Smith et al., 2011): high-arousing negative stimuli were perceived as lasting longer than arousal-matched positive images and low-arousing positive stimuli were perceived as lasting longer than arousal-matched but less positive stimuli (neutral spider pictures).

## Experiment 2: 25% of Positive Images – Spider-Fearful Individuals

In Experiment 2, we sought to establish a baseline response from spider-fearful individuals when judging the duration of emotional images that included a subset of spider images (fear-related stimuli), under conditions of low perceived control. This baseline will serve as a comparison to Experiment 3, in which we increased the level of perceived control over the images experienced by spider-fearful participants. The events were identical to those in Experiment 1. We anticipated temporal distortions to be evident because of the low levels of perceived control. Specifically, we expected high-arousing positive images to be perceived as lasting shorter than both high-arousing negative and spider’s images (i.e., BP for positive images would be larger than the ones for negative and fear-related images).

There is conflicting evidence in the literature regarding how time is experienced when faced with high-arousing threat stimuli compared to high-arousing aversive images. A recent study found that high-arousing negative images representing disgust (e.g., mutilated bodies) were overestimated compared to arousal-matched images representing fear (e.g., attacking animal; Gil and Droit-Volet, 2012). That said, Tipples (2011) showed that fearful and threatening faces were perceived as lasting the same amount of time. However, Tipples found that high levels of fearfulness (as assessed by the Emotionality Activity Sociability Temperament Survey, Buss and Plomin, 1984) were associated with higher overestimations of threat-related facial expressions. This overestimation was driven by participants’ fearfulness and was independent from other personality variables (e.g., level of distress, trait anxiety). Therefore, it appears that fear can have an independent effect on time perception, separate from anxiety.

A critical difference between our study and these past studies was the population tested. Whereas these past studies focused on normal individuals, we are now interested in investigating these effects in a highly reactive population of sub-clinical spider-fearful population. Recent studies have shown that for these individuals, there is an automatic, overlearned aversive response toward spider pictures. For instance, Buetti et al. (2012) investigated rapid reaching movements in spider-fearful individuals who were asked to reach either toward or away from a spider image. In both conditions, participants showed very early avoidance of the spider images in the execution of the reaching response. The initial segment of the reaching trajectory in which the effects were observed is thought to reflect the motor plan assembled prior to movement execution. Therefore, this finding was interpreted as reflecting overlearned, automatic motor responses to fear-related images. Because of the existence of overlearned automatic reactions related to fear-relevant objects compared to other high-arousing negative images (e.g., mutilated bodies), one might expect that spider-fearful participants perceive spider pictures as lasting even longer than arousal-matched negative stimuli, in line with the results of Gil and Droit-Volet (2012). On the other hand, if Tipples (2011) is correct, one might expect that the level of fear of spiders in an individual will be positively correlated with their temporal overestimation of spider images (i.e., negatively correlated with the BP for spider images).

### Methods

#### Participants

Sixteen participants from the same pool as in Experiment 1 participated in Experiment 2. However, this time we tested 16 sub-clinical spider-fearful participants (14 females, 2 males, mean age of 19.9 years, 5.6 of mean at the FSQ).

#### Stimuli, design, and procedure

Stimuli, design, and procedure were the same as in Experiment 1.

### Results

#### Questionnaires and evaluation of the spider pictures

Spider-fearful individuals were very scared of spiders (FSQ = 5.6) and were not at risk for depression (SDQ = 13.3). The level of state anxiety increased from the first to the second assessment, indicating that the images presented in the bisection task affected participant’s anxiety (12.7 vs. 15.8), *t*(15) = −2.13, *p* < 0.05. Participants reported that positive images occurred on 28% of the trials. They considered themselves as being relatively liberal (67%). Importantly, they reported experiencing very low levels of control over the image content (16%).

As expected, spider pictures were evaluated by spider-fearful participants as being highly arousing (mean: 6.5), extremely negative (2.7), and as exerting high domination (3.3). It is important to note that the set of spider images matched the levels of arousal and valence of the IAPS high-arousing negative picture set^3^.

The Pearson’s correlations between the mean arousal, valence, and domination observed at the evaluation of the spider pictures and the mean at the FSQ, indicated that the more spider-fearful participants were afraid of spiders the more they felt aroused (*r* = 0.54, *p* = 0.015) and dominated by the aversive pictures (*r* = −0.48, *p* = 0.029), and the more they evaluated the pictures as being negative (−0.47, *p* = 0.032), which constitutes a good validity check for the FSQ score.

#### Time bisection task

To evaluate whether there was a difference between the three sets of high-arousing images, we ran an ANOVA on the BPs with Image type (IAPS-positive, IAPS-negative, spider) as within-subject factor, remembering that these three groups of images were matched in overall arousal. The results showed a significant main effect, *F*(2,30) = 4.77, *p* < 0.05. The *t*-tests indicated that Spider and IAPS-negative pictures were perceived as lasting the same time (BP of 927 and 912 ms, respectively), *t*(15) = −0.64, *p* = 0.53, but importantly, both Spider and IAPS-negative pictures were perceived as lasting longer than IAPS-positive pictures (BP of 983 ms), *t*(15) = 2.22, *p* < 0.05 and *t*(15) = 3.04, *p* < 0.01, respectively.

Finally, a visual inspection of Figure 2B reveals that the BPs of positive images from the IAPS were almost identical for high- and low-arousing positive images. A *post hoc* comparison confirmed no significant difference between those two conditions (BP of 983 vs. 988 ms, respectively), *t*(15) = −0.20, *p* = 0.85.This *post hoc* finding was unexpected and it indicates that for spider-fearful individuals, perceived duration was only a function of valence (positive vs. negative), with no contribution from arousal. This pattern was also replicated in Experiment 4. The BPs for the four sets of images are shown in Figure 2B.

Finally, we obtained a significant and negative correlation between scores on the FSQ and BPs for the spider picture set (*r* = −0.56, *p* = 0.012), indicating that the more fearful of spiders a participant was, the longer she/he overestimated the duration of spider pictures.

#### Between experiment comparison (Experiment 1 vs. 2)

Participants in Experiments 1 and 2 were exposed to the same sets of stimuli and both saw 25% of highly positive stimuli. However, spider-fearful individuals were faced with a higher percentage of high-arousing negative images than non-anxious individuals (75 vs. 37.5%), half of them representing their object of fear. Thus, overall, the spider-fearful group was exposed to a more stressful experimental context than the non-anxious group.

To ensure that the spider-fearful group only differed with respect to their fear of spiders from the non-anxious group, we compared their responses on the different questionnaires (means and standard errors are shown in Table 1). The *t*-tests clearly indicated that spider-fearful and non-anxious participants only differed on the FSQ (the first group being scared of spiders and the second not), *t*(30) = −14.19, *p* < 0.001 and on their level of state anxiety after performing the time bisection task (spider-fearful participants showed a higher state anxiety than control participants after completing the task), *t*(30) = −3.1, <0.01. The two groups of participants did not differ on the Short Depression Questionnaire, *t*(30) = −0.48, *p* = 0.63, on the first assessment of the State Anxiety Inventory, *t*(30) = −0.92, *p* = 0.36, nor on the DC Scale, *t*(30) = −0.41, *p* = 0.69. Furthermore, at the end of the time bisection task, the two groups reported having been exposed to a similar percentage of positive images, *t*(30) = 0.21, *p* = 0.83, and having experienced similar levels of perceived control over the images, *t*(30) = −0.63, *p* = 0.53. Finally, we also compared their performance on the time bisection task on the two sets of images that were identical across groups: high-arousing positive and high-arousing negative IAPS pictures. When considering these two sets of images, non-anxious and spider-fearful participants showed comparable time distortion effects: an ANOVA on the BPs for IAPS high-arousing positive and negative images with Group as between-subjects factor indicated that neither the main effect of Group was significant (BP of 933 and 948 ms, respectively), *F*(1,30) = 0.11, *p* = 0.75, nor was there an interaction between valence and Group, *F*(1,30) = 0.28, *p* = 0.60.

In spite of the many resemblances between the two groups, in terms of their initial anxiety, as well as in terms of how they reacted to the high-arousing IAPS pictures, we observed a fundamental change in how these two groups judged time: whereas non-anxious participants’ time estimates were sensitive to both arousal and valence, for spider-fearful individuals, time estimates were only a function of the valence of the image (positive images were perceived as shorter than negative images). To anticipate the results of Experiment 4, this difference was not due to the emotional context in the task, as determined by differences in the number of negative images the two groups experienced: in Experiment 4, we found this pattern again, using a very different emotional context.

Thus, we can conclude that the difference in the pattern of results was uniquely due to differences in spider-fearfulness between the two groups.

### Discussion

In Experiment 2 we tested time distortion effects in a highly emotional reactive group of participants under conditions of low levels of perceived of control. Self-report measures of perceived control confirmed that spider-fearful participants experienced a low level of control (16%) in the current experiment. The results from the time bisection task indicated that spider-fearful participants perceived IAPS high-arousing negative images as lasting longer than IAPS high-arousing positive images, just as non-anxious participants did. Supporting previous findings by Tipples (2011), the temporal overestimation observed for IAPS high-arousing negative images was similar to the one observed for high-arousing threat-related images. In addition, the results showed that the more participants were scared of spiders, the longer was the overestimation of the duration of spider images. This result is consistent with Tipples’ results that highly fearful participants overestimate the duration of fearful faces more so than less fearful individuals.

## Experiment 3: 75% of Positive Images – Increased Level of Control

Experiments 1 and 2 provided the general framework to test the hypothesis of most interest to us. We have now established that (a) our spider images give rise to strong temporal distortion effects (both in non-anxious and spider-fearful individuals) and further (b) that the degree of fearfulness toward spiders actually predicts the magnitude of the temporal distortion experienced by spider-fearful individuals. Therefore, our paradigm is robust and sensitive to both the stimulus set that we have chosen and to the personality traits of our participants. The question of interest is then: can a manipulation aimed at increasing the feeling of control in this highly reactive population eliminate the temporal distortion effects observed in Experiment 2? If so, this result would provide strong evidence in support of the hypothesis that perceived control can play an emotion buffering effect and minimize the impact of the emotionality of events on our cognitive processes, as implicitly indexed by the time perception task (Leotti et al., 2010; Mereu and Lleras, in press).

To increase the perceived level of control in our participants, we presented a high proportion of positive images (75%) and a low proportion of negative images (25%) and asked participants to maximize the occurrence of positive images. That is, compared to Experiments 1 and 2, the desired outcome was over-represented in the world’s events, which should lead to a robust feeling of illusory control (Jenkins and Ward, 1965; Alloy and Abramson, 1979). It is important to note that even though we changed the proportion of positive and negative images, we chose the pictures in a way that allowed us to keep the mean arousal and valence comparable across all experiments (see Table 2).

If an increased level of perceived control can buffer the participants’ reactions toward aversive stimuli (Leotti et al., 2010), then one would no longer expect any differences in temporal estimations (i.e., similar BP) across the four image sets.

### Methods

#### Participants

Sixteen (new) spider-fearful participants from the same pool as in Experiment 2 participated in Experiment 3 (16 females, mean age of 19.1 years, 5.6 of mean at the FSQ).

#### Stimuli, design, and procedure

The experimental session was the same as in Experiments 1 and 2. The images from the IAPS and spider images used in Experiment 3 matched the mean arousal and valence of the images presented in Experiments 1 and 2 (see Table 2). See Table 3 for the number of trials per condition. The procedure was the same as in Experiments 1 and 2 with the exception that participants were instructed to maximize the occurrence of positive images. At the end of the session, participants evaluated the spider images using the Self-Assessment Manikin scale (Lang, 1980), starting with the 8 images used in this experiment, and later rating an additional 16 spider images.

**Table 3:** **Number of trials per condition**.

Experiment	Time duration (ms)	IAPS high-arousing positive	IAPS high-arousing negative	IAPS low-arousing positive	Spider pictures
Experiments 1, 2, 5	400	5	15	5	15
	600	5	15	5	15
	800	8	24	8	24
	1000	8	24	8	24
	1200	8	24	8	24
	1400	5	15	5	15
	1600	5	15	5	15
Experiments 3, 4	400	15	5	15	5
	600	15	5	15	5
	800	24	8	24	8
	1000	24	8	24	8
	1200	24	8	24	8
	1400	15	5	15	5
	1600	15	5	15	5

*Note that in Experiments 1, 2, and 5, for the two shortest (400, 600 ms) and longest (1400, 1600 ms) time durations, we randomly selected 15 pictures among the 24 IAPS high-arousing negative pictures as well as 15 pictures among the 24 spider pictures; and for the IAPS high- and low-arousing positive pictures we randomly selected 5 among the 8 pictures from our set. Similarly, for Experiments 3 and 4, for the two shortest and longest time durations, we randomly selected 5 pictures among the 8 IAPS high-arousing negative and spider pictures, and 15 pictures among the 24 IAPS high- and low-arousing positive pictures. Appendix 1 shows the list of the IAPS images used in Experiments 1–5*.

### Results

#### Questionnaires and evaluation of the spider pictures

Scores from the different questionnaires are reported in Table 1. Overall, spider-fearful individuals showed high scores at the FSQ (5.6) and were not at risk for depression (SDQ = 14.4). The level of state anxiety increased from the first to the second assessment (10.4 vs. 13.3), *t*(15) = 2.84, *p* = 0.012. At the end of the time bisection task, spider-fearful participants reported that positive images occurred in 63% of the trials. Participants also considered themselves as being relatively liberal (66%). They evaluated the eight experimental spider pictures as being highly arousing (mean: 6.0), extremely negative (2.2), and as exerting high domination (4.0); and the whole set as being highly arousing (6.7), extremely negative (1.8), and as exerting high domination (3.8). Critically, participants reported sensing a high level of control over the images (54%), as we had expected, which was significantly higher than in Experiments 1 and 2 (*p*s < 0.001).

Unlike Experiment 2, *Pearson’s correlations* no longer showed a significant correlation between participants’ score on the FSQ and any of the ratings of the spider images (arousal, valence, and domination, all *p*s > 0.1). Note that participants rated the images *after* having experienced the images during the experiment and while feeling a high sense of control over the images.

#### Time bisection task

We ran an ANOVA on the BPs measured for high-arousing images with Image type (IAPS-positive, IAPS-negative, spider) as within-subject factor. Unlike Experiment 2, there was no effect of Image type on the magnitude of the BPs, *F*(2,30) = 0.22, *p* = 0.80, see Figure 2C. The BPs for IAPS-positive, IAPS-negative, and spider images were 889, 881, and 866 ms, respectively. The BP for IAPS low-arousing images was 883 ms. As can be seen from the Figure, there was also no difference in BPs between high-arousing positive images and low-arousing positive images (BP of 889 vs. 883 ms), *t*(15) = 0.27, *p* = 0.79.

It is critical to our interpretation of this experiment to measure not only whether the null hypothesis was rejected (it was not), as in traditional Null Hypothesis Significance Testing, but more so, to evaluate the degree of adherence to the null hypothesis. To evaluate this, we used Bayesian statistics (see Masson, 2011), and more specifically, we computed *p*_BIC_: the probability that quantifies the evidence in support of the null hypothesis to any alternative hypothesis, given the data. *p*_BIC_ for Experiment 3 was 0.996, which according to Raftery (1995) provides very strong support in favor of the null hypothesis. In sum, we can confidently conclude that the emotional content of images in Experiment 3 really had no effect whatsoever on time perception.

Finally, in contrast to Experiment 2, *Pearson’s correlations* indicated no significant correlation between the score on the FSQ and the BP observed for the spider pictures set (*p* = 0.21).

### Discussion

The results of Experiment 3 show a substantial contrast with respect to those obtained in Experiment 2. Not only were there no temporal distortion effects across image types obtained, but also, the scores on the Fear of Spider Questionnaire no longer predicted how participants experienced the duration of the spider images during the experiment, nor how they rated the images with the Self-Assessment Manikin scale (Lang, 1980) at the end of the session. Together, these results provide support to our hypothesis that the feeling of control (even if illusory) buffers our cognition (as indexed by performance in the time perception task) from the emotionality of events. Indeed, the effect of control was restricted to a cognitive measure (time judgments): control did not preempt a rise in anxiety during the experiment. This result helps us constraint the boundaries of the buffering effects of perceived control on behavior. That said, these conclusions can only be seen as preliminary given that participants actually experienced more positive images in Experiment 3 than in Experiment 2. Thus, the differences in performance across the two experiments can be ascribed to either the increase in perceived control or the increase in the rate of positive images. Experiment 4 was designed to address this important confound.

## Experiment 4: 75% of Positive Images – Low Level of Control

The aim of this experiment was to rule out the possibility that the absence of an effect of image type on time perception was due to the overall emotional context of the images in Experiment 3. That is, in Experiments 1 and 2, where strong effects of image type on time perception were obtained, the overall rate of positive images was small (25%), whereas in Experiment 3, where no such effects were found, the overall rate of positive images was much higher (75%). Thus, it is possible that when participants find themselves in an overall “positive” context, that image type no longer affects time perception. To rule out this possibility, we re-ran Experiment 3 with a small variation aimed at taking away the sense of control that participants experienced over the images, while maintaining the same high rate of positive images. To do so, participants in this experiment were no longer instructed to try to maximize the occurrence of positive images. Rather, they were told that the computer had an algorithm that was trying to pick as many positive images as possible and that they were to evaluate the computer’s success in this endeavor. They were told that the computer would convey its choice by picking one of two keys and they should simply press the key chosen by the computer. This manipulation was proven to diminish feelings of control in non-anxious individuals on a different study (Mereu and Lleras, in press). As a result, we expected this manipulation to greatly diminish the participant’s sense of control over the events, in spite of the high rate of positive images in the experiment. Because we believe that it is the sense of control (and not the rate of positive images) that was responsible for the absence of time distortions in Experiment 3, we predicted that strong time distortion effects would once again be observed, replicating the results of Experiment 2.

### Methods

#### Participants

Sixteen (new) non-anxious participants from the same pool as in Experiment 1 participated in Experiment 4 (16 females, mean age of 19.5 years, 5.1 of mean at the FSQ) either in exchange for credit in a psychology class (2 participants) or for monetary compensation ($8, 14 participants).

#### Stimuli, design, and procedure

Stimuli, design, and procedures were the same as in Experiment 3. The only difference between Experiments 3 and 4 was the instruction given to participants. Instead of being asked to choose between two keys to try to maximize the occurrence of positive image, participants were instructed to press the key indicated by the computer at the start of each trial. They were told that the computer had an algorithm to try to maximize the occurrence of positive images from a set of positive and negative images and that the key represented the computer’s image choice. So, in this experiment, the trials begun with a display telling them which key to press (“Press the 1 key”; or “Press the 3 key”). The remaining of the instruction were the same as in Experiment 3, with the exception that we asked the following four questions at the end of the time bisection task: (1) How much did the computer choice influence the image content?; (2) Did you feel at any point of the experiment that your choices (instead of those of the computer) influenced the type of images that were presented in the experiment (positive vs. negative images)?; (3) How often did positive images appear?; 4) How liberal are you?

### Results

#### Questionnaires and evaluation of the spider pictures

Our spider-fearful participants scored high at the FSQ (5.1). They were not at risk for depression (SDQ = 13.4). The level of state anxiety was higher in the second than first assessment (STAIS-s = 16.5 vs. 11.8), *t*(15) = −3.61, *p* < 0.01. At the end of the time bisection task, participants reported that about 63% of the images in the experiment were positive^4^. Participants also considered themselves as being relatively liberal (64%). Participants evaluated the eight experimental spider pictures as being highly arousing (mean: 7.3), extremely negative (2.4), and as exerting high domination (2.7); and the whole set as being highly arousing (7.4), extremely negative (2.2), and as exerting high domination (2.9). Critically, they reported that the computer choices were very much responsible for the valence of the images (86%) in the experiment, whereas they themselves felt very little control over the experimental events (11%).

As in Experiment 2, *Pearson’s correlations* between participants’ scores on the FSQ and their ratings of the spider images were related: the higher the score on the FSQ, the higher they rated the mean arousal of the spider images (*r* = 0.45, *p* = 0.040), and the more dominated they felt by the spider images (*r* = −0.51, *p* = 0.023). We did not find a significant correlation between the FSQ scores and the valence score (*r* = 0.13, *p* = 0.31). Finally, although the FSQ score showed once again a negative correlation with the BPs observed for spider images, in this experiment that relationship did not reach significance (*r* = −0.26, *p* = 0.16). It is possible that we failed to find a significant correlation here because of a lack of power.

#### Time bisection task

We ran an ANOVA on the BPs with Image Type (IAPS-positive, IAPS-negative, Spiders, with comparable levels of arousal) as within-subjects factors. The results indicated a significant main effect, *F*(2,30) = 4.99, *p* = 0.013, see Figure 2D. A planned *t*-test confirmed no difference between IAPS high-arousing negative images and high-arousing spider images (BPs 830 and 829 ms), *t*(15) = 0.05, *p* = 0.96. Both sets of high-arousing negative images were temporally overestimated compared to high-arousing positive images (BP 930 ms), *t*(15) = 2.56, *p* < 0.05 and *t*(15) = 2.63, *p* < 0.02, respectively. Interestingly, as in Experiment 2, temporal estimations were similar among high- and low-arousing positive images (BPs 930 and 924 ms), *t*(15) = 0.24, *p* = 0.82.

#### Between experiment comparison (Experiment 2 vs. 4)

A cross-experiment comparison of the two participant groups showed that the two groups were matched in all respects (*p*s > 0.09), except for the reported level of positive images perceived in the experiment, which was significantly higher in Experiment 4 than in Experiment 2 (63 vs. 28%), *t*(30) = 9.10, *p* < 0.001.

With respect to performance on the time bisection task for high-arousing images, we ran an ANOVA on BPs with Image type (IAPS-positive, IAPS-negative, and spider) as within-subjects factors and Experiment as between-subject factor. As before, the results showed that a main effect of Image type, *F*(2,60) = 9.29, *p* < 0.001. More importantly, the main effect of Experiment and the interaction between Experiment and Image type were not significant (*F*s < 0.56). Furthermore, the BPs for the four sets of images did not differ between Experiment 2 and 4 (*p*s > 0.1).

### Discussion

The results of Experiment 4 replicated the results of Experiment 2, in spite of the large difference in the rate of positive images that participants experienced during the time bisection task. That is, in both experiments, spider-fearful participants experienced large time distortion effects that were driven uniquely by the valence of the images: negative images were perceived as lasting longer than positive images, and both sets of positive images were perceived in similar (shorter) fashion, in spite of the large difference in arousal between these two sets of images. We can now confidently conclude that the overall emotional context of the images in an experiment (few vs. many positive images) does not fundamentally alter the direction of the time distortion effects experienced by our participants. More importantly, the results of Experiment 3 can now be more clearly interpreted: we can confidently argue that it was the increase in participants’ feelings of control over the experimental events that eliminated the temporal distortions in that experiment (and not the high rate of positive images).

In sum, we have found strong evidence in favor of the hypothesis that the perception of control over emotional events can have a buffering effect over our cognitive assessments of those events, as indexed here by performance on the time bisection task. That said, this buffering effect did not extend to anxiety: across experiments, and irrespective of the level of control, our spider-fearful participants consistently felt more anxious after being faced with the spider images in the time bisection task, than before. No such increase was found with non-anxious, non-spider-fearful individuals (Experiment 1). Experiment 5 was designed as a final test of the effects of control on the interaction between cognition and emotion.

## Experiment 5: High Level of Control and Cognitive Dissonance

In Experiments 1–3, the participants’ task was consistent with the participants’ general inner goals. That is, participants were asked to minimize negative events or maximize positive events, which are both goals that are compatible with a human being’s general aim of maintaining or moving toward a positive state of wellbeing (e.g., Thorndike, 1933). When participants felt successful in the choice task (Experiment 3), this sense of achievement was compatible with their personal desires of avoiding negative states and aversive events. Under these conditions, their sense of control produced a buffering effect such that the emotionality of events no longer influenced their cognitive decisions in the time bisection task. At first glance, one may want to conclude that *in general* a sense of control will always have a positive effect on our cognition. However, it is easy to design a scenario where participants feel in control over the events, yet, those events lead to a negative internal state. Take for example the instruction: “Try to maximize the occurrence of *negative* images.” Perceived success in this task would lead participants to feel responsible for all the highly aversive images that they are seeing, which presumably, at some level, they do not want to, see. This state of cognitive dissonance may over-ride the otherwise positive effects of perceived control on cognition. Experiment 5 tested this condition in non-anxious, non-spider-fearful participants.

All experimental procedures were identical to those in Experiment 1, with the single exception of the change in instructions. If a sense of control can over-ride the state of cognitive dissonance, one would expect (a) that participants will feel a high level of control and (b) that this simple change in instructions would lead to the absence of time distortion effects (i.e., similar BPs across image sets), even though everything about the procedure and images is identical to Experiment 1, where robust distortion effects were found. If, on the other hand, the state of cognitive dissonance prevents the sense of control from providing some buffering to the participants, then one might expect to replicate Experiment 1 and obtain large distortion effects, in spite of participants having a large sense of control over the experimental events.

### Method

#### Participants

A total of 16 non-anxious participants from the same pool as in Experiment 1 participated in Experiment 5 (16 females, mean age of 19.1 years, 2.1 of mean at the FSQ). We should note that six of our participants had to be replaced. One because she scored too high on the FSQ (5.6) and we wanted to only test non-spider-fearful individuals. Five others were excluded because our instruction manipulation failed to induce a sense of control in them (scores at or near 0%).

#### Stimuli, design, and procedure

Stimuli, design, and procedures were the same as in Experiment 1. The only difference between Experiments 5 and 1 was the instruction given to participants. Instead of being asked to choose between two keys to try to maximize the occurrence of positive image, participants were told to try to *maximize the occurrence of negative events*. Because positive events occurred rarely (25%), we anticipated participants would feel a high level of control over the image content.

### Results

#### Questionnaires and evaluation of the spider pictures

The non-anxious participants were not scared of spiders (FSQ = 2.1) and were not at risk of depression (SDQ = 13.6). The levels of state anxiety were marginally higher during the second than the first assessment (12.9 vs. 11.3), *t*(20) = −1.92, *p* = 0.069. After the time bisection task, participants reported that 26% of the images in the experiment were positive. Participants considered themselves relatively liberal (66%). The evaluation of the spider pictures indicated that they considered the spider images as being low-arousing (3.9), as being neutral (4.8), and as producing moderate feelings of domination (5.5). Critically, participants reported sensing a high level of control over the images (55%).

#### Time bisection task

To test time for distortion effects we ran two separate ANOVAs on BPs with Valence as within-subjects factors and compared arousal-matched conditions (IAPS high-arousing positive images vs. IAPS high-arousing negative images; and IAPS low-arousing positive images vs. low-arousing neutral spider images).

**Figure 3:**
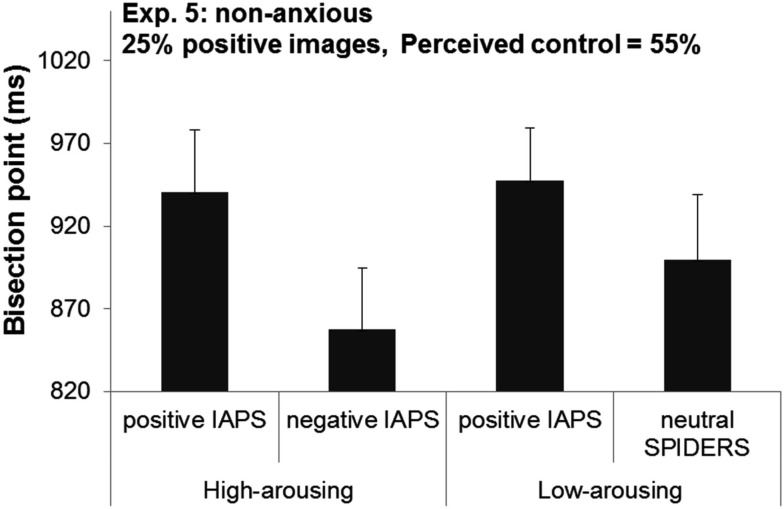
**Bisection points in Experiment 5 as a function of the emotional content of the image events**.

The results indicated that despite the high level of perceived control, high-arousing negative images were still perceived as lasting longer than arousal-matched positive images (BPs 858 vs. 941 ms, respectively), *F*(1,15) = 6.89, *p* = 0.019. On the other hand, BPs did not significantly differ between low-arousing positive and neutral (spiders) images (BPs 948 vs. 899 ms, respectively), *F*(1,15) = 1.71, *p* = 0.21 (see Figure 3). A visual inspection of the results also suggested that we should do a *post hoc* analysis to compare BPs of positive images (high arousal vs. low arousal). Unlike Experiment 1, this comparison revealed no significant difference between these two conditions, *t*(15) = −0.29, *p* = 0.78. In sum, this pattern of results shows that a significant temporal distortion effect was observed only for high-arousing negative IAPS pictures.

#### Between experiment comparison (Experiment 1 vs. 5)

A cross-experiment comparison of the two participant groups, showed that the two groups were matched in all respects (*p*s > 0.42), except for their level of perceived control over the events, which was significantly higher in Experiment 5 (55%) than in Experiment 1 (13%), *t*(30) = 6.24, *p* < 0.001. We ran two ANOVAs on BPs with Valence as within-subjects factors and Experiment as between-subject factor for each level of arousal separately (high and low). For high-arousing images, the results indicated that only the main effect of Valence was significant: negative images were perceived as lasting longer compared to positive images (BPs 881 vs. 951 ms, respectively), *F*(1,30) = 14.49, *p* < 0.001. The main effect of Experiment and the interaction between Valence and Experiment did not reach significance (*F*s < 0.56).

For low-arousing images, the BPs did not differ between Experiments 1 and 5 (BPs 927 vs. 924 ms, respectively) and between positive and neutral images (BPs 922 vs. 928 ms, respectively), *F*s < .0.07, *p*s > 0.79. However, the interaction between Valence and Experiment was significant, *F*(1,30) = 5.79, *p* = 0.022, indicating that participants experienced low arousal images in a different fashion across the two experiments: whereas in Experiment 1, positive images were perceived as lasting longer than negative images, that time distortion effect was not observed in Experiment 5.

### Discussion

The results of Experiment 5 provided a qualified answer to the question: does perceived control help cognition under conditions of cognitive dissonance? We successfully induced a sense of control in our participants, in spite of the cognitive dissonance that they certainly experienced. With this high sense of control, participants experienced temporal distortions in only one condition: high-arousing negative images were perceived as lasting longer than the other image types. In other words, perceived control seemed to inoculate participants from experiencing temporal distortions when viewing low-arousing images. But at high levels of arousal, the valence of the images did alter how subjects experienced them.

Overall, comparing Experiments 1 and 5, the results suggest that experiencing heightened levels of control once again affected the manner in which we processed emotional events. That said, the effects of control were less strong than in Experiment 3, and the critical difference was the goal state of the task: in Experiment 3, participants tried to achieve a positive goal, whereas here, they maximized the occurrence of a negative outcome. Thus the manner in which one elicits control seems to be critical in determining the effect that control will have on cognitive processing, when faced with emotional events. This makes sense because the perception of control is generally considered to be a positive illusion (Taylor and Brown, 1988, 1994), with the adaptive role of moving individuals toward positive moods and states. Thus, the task in Experiment 5 clashes with this adaptive role of experiencing control: asking participants to maximize the number of aversive events puts them in “control” of an environmental situation that is moving them away from a desirable end state (positive mood). From this perspective, it is actually quite remarkable that experiencing control actually had any effects on participants at all.

## General Discussion

The goal of the present study was to test the hypothesis that having a sense of subjective control over experimental emotional events alters the way humans judge the duration of those events. We predicted that having a sense of control would work as an emotional buffer, blocking the effects of emotionality on our cognitive processes (Leotti et al., 2010; Mereu and Lleras, in press). As a strong test of this hypothesis, we chose a population that is very emotionally reactive (sub-clinical spider-fearful individuals) and used fear-related images as stimuli in the time perception task. Our results demonstrated that experiencing a sense of control over the emotional events changed the way these participants judged the duration of those events: whereas in situations of little-to-no control (Experiments 2 and 4), their judgments were influenced by the valence of images (negative images judged to be longer than positive images), once we elicited a sense of control in these participants, the effect of valence on their temporal judgments was eliminated. In fact, when participants experienced little-to-no control, their fearfulness of spiders predicted the time distortion effects, and this correlation was eliminated under conditions of high levels of perceived control.

Two important qualifying results to our conclusions were found. First, whereas the experience of control had a positive effect on cognition (as indexed by performance in the time perception task), it did not change participants’ anxious response to the images: consistently, our spider-fearful participants felt significantly more anxious after the time perception task than before. This finding was robust to differences in the overall emotional contexts of the tasks (in Experiment 2, 75% of images were negative whereas in Experiment 4, 75% were positive) and to the varying sense of control that participants experienced (low control in Experiments 2 and 4, high control in Experiment 3). This finding is consistent with prior findings indicating that the experience of control does not necessarily alter all aspects of our responses to stressing stimuli: the overall estimation of pain and stress is not impacted by the experience of control (Pervin, 1963; Stotland and Blumenthal, 1964; Glass et al., 1969). And whereas control can decrease arousal on the interval preceding the stressful event, no straightforward effect of control has been found on the physiological response to stressors during the stimulation itself (e.g., Corah and Boffa, 1970; Geer et al., 1970; Geer and Maisel, 1972; Gatchel and Proctor, 1976). It is worth remembering that our participants in Experiments 2, 3, and 4 were sub-clinical spider-fearful and thus had a life-long, well established aversion toward spiders. In that sense, it is not a surprise that they consistently reported being more anxious at the end of the experimental session (after being confronted with spider images) than at the beginning. The s-STAI questionnaire (Marteau and Bekker, 1992) that we used is an explicit self-assessment tool to measure state anxiety and therefore it is subject to response biases. As a result, the s-STAI scores may not have been sufficiently sensitive to measure specific responses that participants had to specific spider images throughout the experiment. These phasic anxiety responses may have been modulated by perceived control on a trial by trial basis, but we had no way of measuring such effects. Follow-up studies using different anxiety measuring techniques as well as physiological arousal measures are warranted to better assess the impact of perceived control on anxiety. On the positive side, the time perception task represents an implicit measure of the effects of emotional stimuli on our participants’ cognitive system. Participants were not asked directly to judge or respond to the emotionality of each image in the task, they simply reported whether the image duration seemed closest to the shorter or to the longer standards. This implicit measure successfully showed an effect of perceived control on the cognitive task of time estimation.

A second qualifying result to our overall conclusion that a sense of control can protect our cognitive processes (here, time judgments) from the usual effects of emotional stimuli relates to the conditions in which the sense of control is obtained. Given that, in our experiments, eliciting a sense of control depends on asking participants to exert a choice, the choice must be one that seeks to obtain an outcome aligned with participants’ inner sense of wellbeing. When participants are asked to seek goals that run counter to their wellbeing (Experiment 5), the effects of control are diminished and the emotionality of events does seem to end up altering cognition (at least for high-arousing stimuli). Put simply, the experience of control is not a magic bullet: simply asking participants to play an active role in emotional situations will not ensure that emotions will not influence their cognitive mechanisms.

### Time perception

Models of time perception differ on whether they posit the existence of an internal clock (or pacemaker) that can be sampled to estimate time (e.g., Gibbon, 1977; Gibbon et al., 1984) or whether they do without an internal clock (Church and Broadbent, 1990; Matell and Meck, 2004). Though the existence of an internal clock is a matter of debate (see Gorea, 2011), the vast majority of the literature on time perception uses the SET model which does incorporate an internal clock (Gibbon et al., 1984). Within this framework, temporal distortions are easily modeled: they can arise because the internal clock has changed its ticking rate (typically, an effect ascribed to manipulations of arousal) or because the extent to which one can attend to the counting process itself has been manipulated. That is, if we seldom pay attention to the counting process, then time will appear to have gone quickly (few samples were taken), if we often pay attention to the counting process, then time will appear to slow down (many tic samples were taken).

Droit-Volet and colleagues (e.g., Droit-Volet et al., 2004; Droit-Volet and Meck, 2007; Droit-Volet and Gil, 2009) have published substantially on the topic of how emotions affect our estimation of time. The results obtained in this literature are as follows: for high-arousing events, negative events are perceived as lasting longer than positive events because of attentional avoidance. When faced with highly aversive stimuli, our attention quickly turns inward, and the counting process begins quickly. In contrast, high-arousing positive events do not cause this quick avoidance reaction, and thus less attention is available for time estimation. In contrast, for low-arousing images, the effects are reversed. Negative images actually tend to capture attention. But, given the absence of threat in the environment, there is no need to quickly disengage from the image, which delays attention to the counting process. More recent studies have started to better differentiate different types of aversive stimuli: fear, disgust, threat are now being considered independently (Tipples, 2011; Gil and Droit-Volet, 2012).

Within the framework proposed by Droit-Volet et al. (2004) the effect of perceived control on time perception fits best with an “attentional locus.” It is as if, under high levels of perceived control in Experiment 3, participants can better engage and disengage from the images, or at least do so in a similar fashion across all image types. Perhaps, success in the control task allows participants to focus on a positive aspect of their ongoing experience (their success), allowing them to be less vulnerable to the attentional pull of emotional events. We hasten to add that the task we used to induce the feeling of control (“maximize events of type X”) was the same across all experiments. Thus, it is not the inclusion of this new task that changed the attentional engagement to the various images, but the perceived level of success in that task. Further, when control was high, but the experience was not positive (Experiment 5), perceived control did not fully protect participants against temporal distortions. Overall, it is possible that the “buffering” effect proposed by Leotti et al. (2010) is attentional in nature: when we feel in control, we might attend differently to world events. This issue is now the focus of investigation in our laboratory.

Our results provide an important contribution to the time perception literature. First, Experiment 1 replicated the standard modulations of time judgments by valence and arousal (Angrilli et al., 1997; Smith et al., 2011). Furthermore, the results of Experiments 2 and 4 provide additional support regarding the stability of these effects: the overall emotional context of a task does not impact the specific effect of image type on BPs. That is, both when negative events were frequent and when they were rare, the valence of the image being judged on a given trial determined whether this image would be perceived as relatively longer or shorter than other image types in that experiment. Another important result from our experiments was the replication of Tipples’ (2011) observation that individual differences in fearfulness predict differences in time perception: the more fearful participants were of spiders, the longer they perceived the duration of spider images. This finding was obtained when participants felt little-to-no control over experimental events.

Finally, the results of Experiments 2 and 4 represent a new departure from the literature on one important point: we twice failed to, see an effect of arousal on the perceived duration of positive images. That is, whereas it is typically observed that highly arousing positive images are perceived as lasting a short time and low-arousing positive images as lasting a relatively longer period of time, here we found no such difference. In fact, our results suggested that our participants’ perception of time was entirely driven by valence, with positive events being judged as lasting shorter than both aversive and threatening events. We can speculate that our participants were probably highly aroused throughout the experiment because of the anticipation of having to face spider pictures (in an unpredictable fashion). Thus, perhaps *all* events were perceived as occurring in a state of high arousal. If that is true, then that would explain why only an effect of valence was found.

### Possible limitations of the present findings

There are several potential limitations to this study that should be acknowledged. First, with respect to the selection of stimuli, McGraw et al. (2010) recently published a study showing that bipolar scales for measuring emotional valence (as it is done in the IAPS ratings) are inappropriate because they do not reflect the psychological difference in intensity between positive and negative emotions. That is, a score of 3 in the IAPS valence scale is 2 units away from the neutral point in the scale (5), but the intensity of this negative emotion is larger than the intensity of a positive image with a score of 7. Thus, it is possible that our group of negative images were therefore psychologically more intense than the groups of positive images. Fortunately, the main point of the current paper does not rest on perfectly equating valences across image sets. Rather, our design and experimental logic lay on the direct comparison of performance by participants looking at the exact same images (see comparison between Experiments 3 and 4). Thus, the key in the logic is that emotional images that usually produce distortions in time perception (as we verified in Experiments 1, 2, and 4) failed to do so when one judges them under conditions of high perceived control (Experiment 3).

The between-subject structure of our design also presents limitations to the current findings. Although we successfully equalized the participant groups across experiments, experiments were run at different times during the semester and some participants received monetary compensation for participation. Thus, one cannot be entirely certain that there were not “unmeasured” differences between groups. To assuage these concerns, we should point out that our results replicated previous results in the literature (Experiment 1 replicates Angrilli et al., 1997; Smith et al., 2011), and also replicated within this study (Experiments 2 and 4). Thus, we can feel confident that our results are robust and replicable. We should also note that the null result we obtained in Experiment 3 should be interpreted with a measure of caution. Even though we (a) predicted a null result, (b) replicated a null result from a different study (Mereu and Lleras, in press), and (c) provided a quantitative measure to estimate the adherence of the null hypothesis (Masson, 2011), one can never be certain as to the reasons why a null result is found in an experiment.

### Conclusion

In this manuscript, we studied one way in which emotions can impact cognition, specifically, how the emotional content of an event alters how we estimate the duration of that event. Such time distortions have been interpreted as a negative effect of emotions on cognitive processing: the emotionality of an event alters the manner in which our attention system engages in the task of counting time. Here, we demonstrated that the manner in which participants engage with the stimuli can significantly modulate this deleterious effect of emotions on cognitive processing: when participants feel a high degree of control over the emotionality of events in the experiment, they seem to better control their attention system, such that the emotionality of the image no longer impacts their cognitive assessment of that image. Given that in most if not all current experiments investigating the effects of emotion on cognition, participants are put into a situation where they have no control over the emotional events in the experiment, our results indicate that it may be worthwhile and fruitful to study the impact of emotion on cognition in situations where participants can feel some degree of control over the emotional events.

## Conflict of Interest Statement

The authors declare that the research was conducted in the absence of any commercial or financial relationships that could be construed as a potential conflict of interest.

## Footnotes

^1^The full set of spider images can be requested by contacting the corresponding author.

^2^Overall, in Experiment 1, participants were shown 37.5% of high-arousing negative images, 37.5% of low-arousing neutral images, 12.5% of high-arousing positive images, and 12.5% of low-arousing positive images.

^3^Overall, spider-fearful participant were shown 25% of highly positive images (12.5% being low-arousing and 12.5% high-arousing) and 75% of high-arousing negative images.

^4^Overall participants saw 37.5% of IAPS high-arousing positive images, 37.5% of IAPS low-arousing positive images, 12.5% of IAPS high-arousing negative images and 12.5% of high-arousing threatening images.

## Appendix

List of the IAPS images (Lang et al., 2008) used in Experiments 1–5.

In *Experiments 1, 2, and 5* we chose 8 low-arousing positive pictures (1441, 1450, 1600, 2057, 5030, 5040, 5210, 5725), 8 high-arousing positive pictures (4607, 4647, 4649, 4651, 4652, 4656, 4668, 4670), and 24 high-arousing negative pictures (3000, 3001, 3010, 3030, 3053, 3061, 3062, 3063, 3064, 3068, 3071, 3101, 3103, 3120, 3131, 3140, 3150, 3168, 3185, 3190, 3191, 3225, 3250, 3261).

In *Experiments 3–4* we chose 24 low-arousing positive pictures (1333, 1419, 1441, 1450, 1600, 1602, 1620, 2040, 2057, 2060, 2153, 2302, 2304, 2306, 2358, 2388, 5001, 5030, 5040, 5210, 5725, 5764, 5831, 7325), 24 high-arousing positive pictures (4607, 4608, 4611, 4631, 4647, 4649, 4651, 4652, 4656, 4664, 4668, 4670, 5621, 5629, 7405, 8033, 8080, 8179, 8180, 8185, 8186, 8370, 8490, 8492), 8 high-arousing negative pictures (3000, 3001, 3010, 3030, 3061, 3062, 3150, 3168).
